# Crystal structure and Hirshfeld surface analysis of methyl 4-[(*E*)-2-(5-bromo-2-meth­oxy­benzyl­idene)hydrazin­yl]-3-nitro­benzoate

**DOI:** 10.1107/S2056989018011325

**Published:** 2018-08-14

**Authors:** Tanvirbanu J. Malek, Sahaj A. Gandhi, Vijay Barot, Mukesh Patel, Urmila H. Patel

**Affiliations:** a LDRP–Institute of Technology & Research, Kadi Sarva Vishwavidhayalay, Gandhinagar, India; b Bhavan’s Shri I. L. Pandya Arts –Science and Smt. J. M. Shah Commerce College, Dakor, Gujarat, India; c P.G. Center in Chemistry, Smt. S. M. Panchal Science College, Talod, India; d Department of Physics, Sardar Patel University, Vallabh Vidyanagar, India

**Keywords:** crystal structure, hydrazine derivative, graph set motif, hydrogen bond, Hirshfeld surface analysis

## Abstract

The title compound, C_16_H_14_BrN_3_O_5_, is a novel halogen (Br) substituted hydrazine derivative. The hydrazine derivatives were the group of compounds with the general structure, *R*_1_ *R*_2_C=NNH_2_ (Uppal *et al.*, 2011 ▸), with the central *R*C=NNH_2_ moiety bridging two different groups on both sides. An all-*trans* configuration of the backbone (*R*C=NNH_2_) results in an extended mol­ecular conformation. The dihedral angle between the 5-bromo-2-meth­oxy­phenyl ring and the nitrophenyl ring is 4.4 (3)°. Intra­molecular N—H⋯O inter­actions form *S*(6) graph-set motifs, while C—H⋯O and C—H⋯N inter­actions form *S*(5) graph-set motifs. Symmetry-related mol­ecules are linked by C—H⋯O inter­molecular inter­actions forming an *R*_2_^1^(10) graph-set motif. There are nearly face-to-face directional specific π–π stacking inter­actions between the centroids of the nitrophenyl ring and the benzene ring of the 5-bromo-2-meth­oxy group [centroid–centroid distance = 3.6121 (5) Å and slippage = 1.115 Å], which also contributes to the mol­ecular packing. The Hirshfeld surface analysis was performed in order to visualize, explore and qu­antify the inter­molecular inter­actions in the crystal lattice of the title compound.

## Abstract 2

The title compound is a novel halogen-substituted hydrazine derivative. Intra­molecular N—H⋯O inter­actions form *S*(6) graph-set motifs, while C—H⋯O and C—H⋯N inter­actions form *S*(5) graph-set motifs.

## Chemical context   

Hydrazine and its derivatives have attracted much attention due to their synthetic potential for organic and inorganic chemical reactions and diverse useful properties (Levrand *et al.*, 2007 ▸; Li *et al.*, 2011 ▸). Hydrazine-based coupling methods are used in medical biotechnology to couple drugs to targeted anti­bodies, *e.g.* anti­bodies against a certain type of cancer cell (Wu *et al.*, 2005 ▸). Hydrazine possesses diverse biological and pharmacological properties, such as anti­microbial, anti-inflammatory, analgesic, anti­fungal, anti­tubercular, anti­viral, anti­cancer, anti­platelet, anti­malarial, anti­convulsant, cardio-protective, anti­helmintic, anti­protozoal (Rollas & Küçükgüzel, 2007 ▸), anti­trypanosomal and anti­schistosomiasis (Narang *et al.*, 2012 ▸). These compounds contain a C=N bond, which is conjugated with a lone pair of electrons of the functional N atom (Corey & Enders, 1976 ▸). The N atom of the hydrazine is nucleophilic and the C atom has both an electrophilic and a nucleophilic nature (Corey & Enders, 1976 ▸). The α-hydrogen of hydrazine is more potent than that of acidic ketones (Belskaya *et al.*, 2010 ▸). The combination of hydrazine with other functional groups results in new compounds with unique physical and chemical characteristics (Xavier *et al.*, 2012 ▸). Owing to their biological and pharmacological properties, hydrazine derivatives play an important role for the synthesis of heterocyclic compounds (Banerjee *et al.*, 2009 ▸).
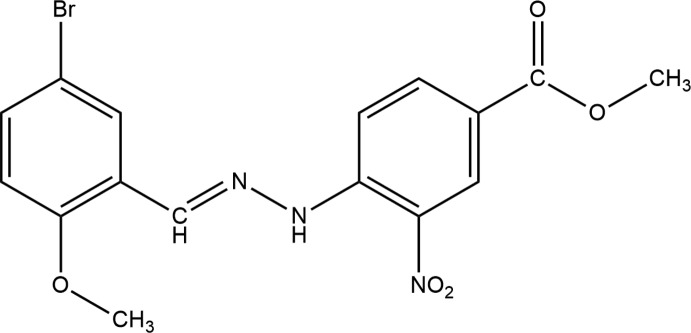


## Structural commentary   

**Figure 1:**
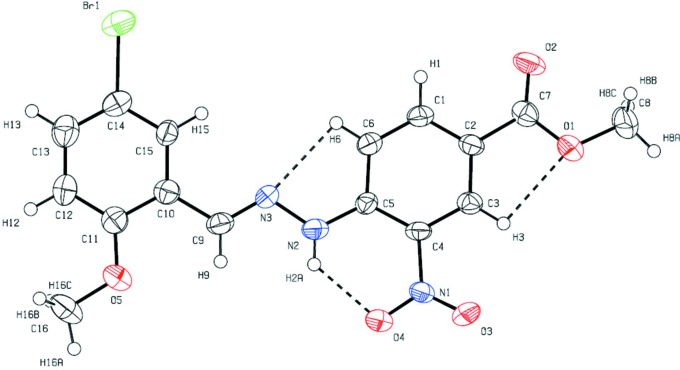
The mol­ecular structure of the title compound with the atom-labelling scheme. Displacement ellipsoids are drawn at the 50% probability level.

Fig. 1 ▸ displays the title mol­ecule with the atom-labelling scheme. Intra­molecular N2—H2*A*⋯O4 inter­actions form *S*(6) graph-set motifs and C3—H3⋯O1 and C6—H6⋯N3 inter­actions form *S*(5) graph-set motifs. The central bridging moiety R_2_C=NNHR_1_ adopts an all-*trans* conformation about the C10—C9, C9—N3, N3—N2 and N2—C5 bonds, with torsion angles of 176.0 (6), −178.1 (5), −177.0 (6) and 173.6 (6)°, leading to an extended mol­ecular conformation, thereby causing the terminal bromo­meth­oxy­phenyl ring and nitro­phenyl­ring to occupy almost the same plane; the dihedral angle between the rings is 4.4 (3)°.

## Supra­molecular features and Hirshfeld surface analysis   

**Figure 2:**
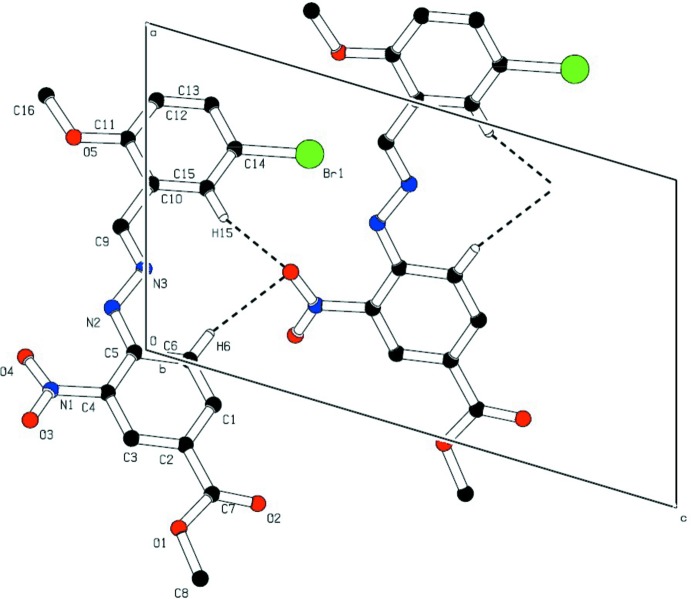
A view of part of the crystal structure of the title compound, showing the formation of C—H⋯O hydrogen bonds (dashed bonds).

A significant number of weak C—H⋯O, C—H⋯N and N—H⋯O intra­molecular inter­actions and C—H⋯O inter­molecular inter­actions (Table 1 ▸), along with direction-specific nearly face-to-face π–π stacking inter­actions, are responsible for the stability of the mol­ecular packing. Inter­molecular C—H⋯O hydrogen-bond inter­actions forming 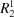
(10) ring (Fig. 2 ▸). There are nearly face-to-face direction-specific π–π stacking inter­actions between the centroids of the nitrophenyl ring (*x*, *y*, *z*) and the benzene ring of the 5-bromo-2-meth­oxy group (*x* − 1, *y*, *z*) [centroid–centroid distance = 3.6121 (5) Å and slippage = 1.115 Å], which also contributes to the mol­ecular packing. The Br atom does not take part in any inter­actions. The nearest Br⋯C7(−*x* + 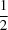, *y* − 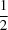, −*z* + 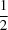) distance in the mol­ecular structure is 3.6112 (7) Å.

**Figure 3:**
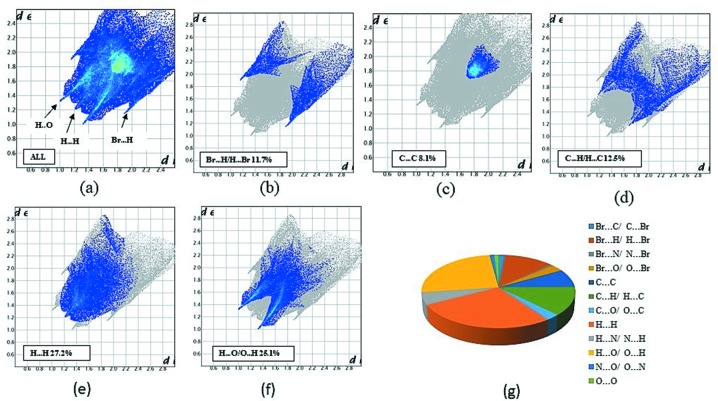
The full two-dimensional fingerprint plots, and those delineated into (*a*) all inter­actions (*b*) Br⋯H, (*c*) C⋯C, (*d*) C⋯H/H⋯C, (*e*) H⋯H and (*f*) H⋯O/O⋯H contacts showing the percentages of contacts contributed to the total Hirshfeld surface area. (*g*) Pi chart.

Hirshfeld surface analysis serves as a powerful tool for gaining additional insight into inter­molecular inter­actions of mol­ecular crystals. The Hirshfeld surfaces are mapped with 2D fingerprint plots presented using *CrystalExplorer3.1* and it provides a summary of the inter­molecular contacts in the crystal (McKinnon *et al.*, 2004 ▸; Spackman & Jayatilaka, 2009 ▸). The 2D fingerprint plots (Fig. 3 ▸) show that the inter­molecular H⋯H and O⋯H inter­actions dominate and complement the Hirshfeld surfaces. The fingerprint plots can also be decomposed to highlight particular atom-pair close contacts (Luo *et al.*, 2013 ▸) and enables separation of contributions from different inter­action types. Two sharp spikes pointing towards the upper left of the plot are typical C—H⋯O hydrogen bonds. This portion corresponds to H⋯O inter­actions comprising 25.1% of the total Hirshfed surfaces. Two sharp spikes pointing towards the lower left of the plot are typical Br⋯H hydrogen bonds. This portion corresponds to Br⋯H inter­actions comprising 11.7% of the total Hirshfeld surfaces. The broad region bearing short and narrow spikes at the middle of plot is reflected as H⋯H inter­action comprising 27.2% of the total Hirshfeld surfaces. Apart from these, the presence of Br⋯C, Br⋯N, Br⋯O, C⋯O, H⋯N, N⋯O and O⋯O inter­actions were observed (Pi chart; Fig. 4*g*), which are summarized in Table 2 ▸ (Li *et al.*, 2013 ▸; Luo & Sun, 2014 ▸; Seth *et al.*, 2011 ▸).

## Database survey   

While searching for 2-phenyl­hydrazine in the Cambridge Structural Database (CSD, Version 53.7; Groom *et al.*, 2016 ▸), four significant structures were found [CSD refcodes AYSOD (Tahir *et al.*, 2011 ▸), DUSBID (Mufakkar *et al.* 2010 ▸), DUSNUB (Shad *et al.* 2010 ▸) and DUSNUB01 (Toledano-Magaña *et al.*, 2015 ▸)]. Also, the crystal structure of the unsubstituted phenyl hydrazine has been reported in the CSD [ZZZGWW02 (Vickery *et al.*, 1985 ▸) and ZZZGWW03 (Günes, *et al.*, 2003 ▸)]. The two phenyl rings in AYSOD (two mol­ecules in the asymmetric unit), DUSBID and DUSNUB (two mol­ecules in the asymmetric unit) are inclined to each other by 2.44 (18) and 14.08 (19)° (in mol­ecules *A* and *B*), 9.30 (6)°, and 13.01 (10) and 14.05 (10)° (in mol­ecules *A* and *B*), respectively, compared to 4.4 (3)° in the title compound. The crystal packing of the two compounds is significantly different. In AYSOD, N—H groups do not form hydrogen bonds, in DUSBID, the mol­ecules are linked by N—H⋯π inter­actions, and in DUSNUB, both mol­ecules form inversion dimers linked by pairs of N—H⋯O hydrogen bonds, thereby generating 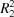
(16) motif rings (Bernstein *et al.*, 1995 ▸). In the title compound, intra­molecular N—H⋯O and only inter­molecular C—H⋯O hydrogen bonds are present; there are no C—H⋯π inter­actions. Very few similar hydrazine derivatives are reported in the literature (Cortés *et al.*, 2013 ▸; Dey & Chopra, 2017 ▸). In those crystal structures, a halogen group (Cl and F, respectively) is present, while in this crystal structure, Br is present.

## Synthesis and crystallization   

The title compound was synthesized in one step by heating the hydrazine derivative 3-nitro­benzohydrazide (0.181 mg) with a slight excess of 5-bromo-2-meth­oxy­benzaldehyde (0.215 mg) in an acetic acid solution (10 ml). The reaction mixture was refluxed for 8 h. The solid product formed during reflux was filtered off, washed and dried over anhydrous calcium chloride in a vacuum desiccator (yield 75%). The final product was soluble in acetone, dimethyl sulfoxide (DMSO), di­methyl­formamide (DMF), methanol, ethanol and ethyl acetate, *etc*. Transparent orange-coloured needle-shaped diffraction-quality single crystals of the title compound were grown by slow evaporation using methanol as the solvent at room temperature.

## Refinement   

Crystal data, data collection and structure refinement details are summarized in Table 3 ▸. The coordinates of the H atoms of the N2—H2 and C9—H9 groups were refined [N2—H2 = 0.83 (6) Å and C9—H9 = 0.90 (5) Å]. Other H atoms were placed in geometrically idealized positions and constrained to ride on their parent atoms, with C—H = 0.93–0.97 Å, and refined as riding with *U*_iso_(H) = *xU*_eq_(C), where *x* = 1.5 for methyl and *x* = 1.2 for all other H atoms.

## Supplementary Material

Crystal structure: contains datablock(s) I. DOI: 10.1107/S2056989018011325/dx2007sup1.cif

Structure factors: contains datablock(s) I. DOI: 10.1107/S2056989018011325/dx2007Isup2.hkl

Supporting information file. DOI: 10.1107/S2056989018011325/dx2007Isup3.cml

CCDC reference: 1860856

Additional supporting information:  crystallographic information; 3D view; checkCIF report

## Figures and Tables

**Table 1:** Hydrogen-bond geometry (Å, °)

*D*—H⋯*A*	*D*—H	H⋯*A*	*D*⋯*A*	*D*—H⋯*A*
N2—H2*A*⋯O4	0.83	2.03	2.635 (3)	129
C3—H3⋯O1	0.93	2.39	2.712 (4)	100
C6—H6⋯N3	0.93	2.40	2.731 (4)	101
C6—H6⋯O4^i^	0.93	2.59	3.444 (5)	152
C15—H15⋯O4^i^	0.93	2.46	3.358 (4)	161

Symmetry code: (i) 
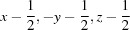
.

**Table 2:** Summary of the various contacts and their contributions to the Hirshfeld surface

Contacts	Percentage contribution
Br⋯C/C⋯Br	1.6
Br⋯H/H⋯Br	11.7
Br⋯N/N⋯Br	0.7
Br⋯O/O⋯Br	2.8
C⋯C	8.1
C⋯H/H⋯C	12.5
C⋯O/O⋯C	2.7
H⋯H	27.2
H⋯N/N⋯H	5.5
H⋯O/O⋯H	25.1
N⋯O/O⋯N	1.1
O⋯O	1.0

**Table 3:** Experimental details

Crystal data	
Chemical formula	C_16_H_14_BrN_3_O_5_
*M*_r_	408.21
Crystal system, space group	Monoclinic, *P*2_1_/*n*
Temperature (K)	293
*a*, *b*, *c* (Å)	8.3262 (11), 14.8369 (19), 14.0764 (13)
β (°)	106.558 (14)
*V* (Å^3^)	1666.8 (4)
*Z*	4
Radiation type	Mo *K*α
μ (mm^−1^)	2.50
Crystal size (mm)	0.09 × 0.08 × 0.06

Data collection	
Diffractometer	Bruker APEXII CCD
Absorption correction	Multi-scan (North *et al.*, 1968 ▸)
*T*_min_, *T*_max_	0.666, 1.000
No. of measured, independent and observed [*I* > 2σ(*I*)] reflections	4830, 3187, 1726
*R*_int_	0.065
(sin θ/λ)_max_ (Å^−1^)	0.682

Refinement	
*R*[*F* ^2^ > 2σ(*F* ^2^)], *wR*(*F* ^2^), *S*	0.096, 0.202, 1.09
No. of reflections	3187
No. of parameters	235
H-atom treatment	H atoms treated by a mixture of independent and constrained refinement
Δρ_max_, Δρ_min_ (e Å^−3^)	0.66, −0.69

Computer programs: *APEX2* and *SAINT* (Bruker, 2008 ▸), *SHELXS97* and *SHELXL97* (Sheldrick, 2008 ▸) and *PLATON* (Spek, 2009 ▸).

## supplementary crystallographic information

### Crystal data

**Table:**

C_16_H_14_BrN_3_O_5_	*F*(000) = 824
*M**_r_* = 408.21	*D*_x_ = 1.627 Mg m^−^^3^
Monoclinic, *P*2_1_/*n*	Mo *K*α radiation, λ = 0.71073 Å
*a* = 8.3262 (11) Å	Cell parameters from 1261 reflections
*b* = 14.8369 (19) Å	θ = 3.3–23.2°
*c* = 14.0764 (13) Å	µ = 2.50 mm^−^^1^
β = 106.558 (14)°	*T* = 293 K
*V* = 1666.8 (4) Å^3^	Plate, yellow
*Z* = 4	0.09 × 0.08 × 0.06 mm

### Data collection

**Table:**

Bruker APEXII CCD diffractometer	1726 reflections with *I* > 2σ(*I*)
Radiation source: sealed tube	*R*_int_ = 0.065
φ and ω scans	θ_max_ = 29.0°, θ_min_ = 3.6°
Absorption correction: multi-scan (North *et al.*, 1968)	*h* = −10→11
*T*_min_ = 0.666, *T*_max_ = 1.000	*k* = −19→10
4830 measured reflections	*l* = −9→18
3187 independent reflections	

### Refinement

**Table:**

Refinement on *F*^2^	Hydrogen site location: mixed
Least-squares matrix: full	H atoms treated by a mixture of independent and constrained refinement
*R*[*F*^2^ > 2σ(*F*^2^)] = 0.096	*w* = 1/[σ^2^(*F*_o_^2^) + (0.0381*P*)^2^ + 5.1653*P*] where *P* = (*F*_o_^2^ + 2*F*_c_^2^)/3
*wR*(*F*^2^) = 0.202	(Δ/σ)_max_ < 0.001
*S* = 1.09	Δρ_max_ = 0.66 e Å^−^^3^
3187 reflections	Δρ_min_ = −0.69 e Å^−^^3^
235 parameters	Extinction correction: SHELXL97 (Sheldrick, 2008), Fc^*^=kFc[1+0.001xFc^2^λ^3^/sin(2θ)]^-1/4^
0 restraints	Extinction coefficient: 0.0076 (10)

### Special details

**Table:**

Geometry. All esds (except the esd in the dihedral angle between two l.s. planes) are estimated using the full covariance matrix. The cell esds are taken into account individually in the estimation of esds in distances, angles and torsion angles; correlations between esds in cell parameters are only used when they are defined by crystal symmetry. An approximate (isotropic) treatment of cell esds is used for estimating esds involving l.s. planes.

### Fractional atomic coordinates and isotropic or equivalent isotropic displacement parameters (Å^2^)

**Table:**

	*x*	*y*	*z*	*U*_iso_*/*U*_eq_	
C1	0.3935 (9)	0.2162 (5)	0.6270 (5)	0.038 (2)	
H1	0.415174	0.223200	0.695123	0.046*	
C2	0.2457 (9)	0.1749 (5)	0.5739 (5)	0.0348 (19)	
C3	0.2169 (9)	0.1645 (5)	0.4728 (5)	0.0339 (18)	
H3	0.118584	0.137270	0.435409	0.041*	
C4	0.3340 (9)	0.1944 (5)	0.4272 (5)	0.0328 (18)	
C5	0.4810 (9)	0.2391 (5)	0.4784 (5)	0.0308 (17)	
C6	0.5083 (9)	0.2468 (6)	0.5818 (5)	0.040 (2)	
H6	0.606744	0.273342	0.620051	0.049*	
C7	0.1197 (10)	0.1486 (6)	0.6245 (6)	0.0386 (19)	
C8	−0.1484 (10)	0.0834 (7)	0.6023 (6)	0.061 (3)	
H8A	−0.236862	0.055874	0.551367	0.091*	
H8B	−0.107222	0.041302	0.655468	0.091*	
H8C	−0.190635	0.135968	0.626904	0.091*	
C9	0.8528 (10)	0.3310 (6)	0.4527 (5)	0.040 (2)	
C10	1.0108 (9)	0.3711 (6)	0.5111 (5)	0.0373 (19)	
C11	1.1267 (10)	0.4052 (5)	0.4648 (6)	0.040 (2)	
C12	1.2709 (10)	0.4470 (6)	0.5203 (6)	0.046 (2)	
H12	1.345065	0.471252	0.488742	0.055*	
C13	1.3069 (10)	0.4532 (6)	0.6226 (6)	0.052 (2)	
H13	1.404718	0.480982	0.659877	0.063*	
C14	1.1940 (10)	0.4174 (6)	0.6681 (5)	0.043 (2)	
C15	1.0485 (9)	0.3762 (5)	0.6140 (5)	0.0357 (18)	
H15	0.975310	0.351682	0.646202	0.043*	
C16	1.1856 (12)	0.4401 (7)	0.3120 (6)	0.069 (3)	
H16A	1.141419	0.428322	0.242387	0.104*	
H16B	1.185656	0.503869	0.323555	0.104*	
H16C	1.298062	0.417511	0.335016	0.104*	
N1	0.2911 (8)	0.1797 (5)	0.3208 (4)	0.0370 (16)	
N2	0.5978 (8)	0.2714 (5)	0.4364 (5)	0.0424 (18)	
N3	0.7457 (8)	0.3049 (5)	0.4962 (4)	0.0393 (16)	
O1	−0.0146 (7)	0.1088 (4)	0.5621 (4)	0.0494 (16)	
O2	0.1325 (7)	0.1613 (4)	0.7107 (4)	0.0548 (17)	
O3	0.1809 (8)	0.1260 (5)	0.2817 (4)	0.0612 (18)	
O4	0.3693 (6)	0.2228 (4)	0.2728 (3)	0.0544 (17)	
O5	1.0838 (7)	0.3963 (4)	0.3644 (4)	0.0545 (17)	
BR1	1.24638 (13)	0.41918 (8)	0.80847 (6)	0.0681 (5)	
H2A	0.576 (7)	0.270 (4)	0.375 (4)	0.014 (16)*	
H9	0.825 (7)	0.329 (4)	0.386 (4)	0.012 (15)*	

### Atomic displacement parameters (Å^2^)

**Table:**

	*U*^11^	*U*^22^	*U*^33^	*U*^12^	*U*^13^	*U*^23^
C1	0.037 (4)	0.050 (6)	0.025 (3)	0.001 (4)	0.004 (4)	0.002 (4)
C2	0.035 (4)	0.046 (5)	0.025 (3)	0.004 (4)	0.011 (3)	0.004 (3)
C3	0.033 (4)	0.040 (5)	0.028 (3)	0.005 (4)	0.007 (3)	0.004 (3)
C4	0.038 (4)	0.040 (5)	0.020 (3)	0.002 (4)	0.008 (3)	−0.003 (3)
C5	0.025 (4)	0.039 (5)	0.028 (3)	0.001 (4)	0.006 (3)	−0.003 (3)
C6	0.033 (4)	0.056 (6)	0.030 (3)	−0.003 (4)	0.006 (4)	−0.005 (4)
C7	0.038 (4)	0.041 (6)	0.039 (4)	0.008 (4)	0.016 (4)	0.004 (4)
C8	0.038 (5)	0.091 (8)	0.059 (5)	−0.003 (5)	0.022 (4)	0.007 (5)
C9	0.037 (5)	0.051 (6)	0.027 (4)	0.002 (4)	0.004 (4)	−0.001 (4)
C10	0.032 (4)	0.044 (5)	0.035 (4)	0.007 (4)	0.008 (4)	0.003 (4)
C11	0.035 (4)	0.040 (6)	0.042 (4)	0.006 (4)	0.010 (4)	0.004 (4)
C12	0.039 (5)	0.042 (6)	0.056 (5)	−0.008 (4)	0.012 (4)	−0.001 (4)
C13	0.039 (5)	0.055 (6)	0.060 (5)	0.000 (4)	0.011 (5)	0.003 (5)
C14	0.044 (5)	0.042 (6)	0.037 (4)	0.003 (4)	0.002 (4)	−0.001 (4)
C15	0.031 (4)	0.037 (5)	0.038 (4)	−0.006 (4)	0.007 (4)	−0.003 (4)
C16	0.073 (6)	0.095 (9)	0.052 (5)	−0.006 (6)	0.037 (5)	0.010 (5)
N1	0.034 (4)	0.053 (5)	0.025 (3)	0.001 (3)	0.011 (3)	0.003 (3)
N2	0.038 (4)	0.064 (5)	0.025 (3)	−0.002 (4)	0.008 (3)	−0.004 (3)
N3	0.033 (4)	0.053 (5)	0.030 (3)	−0.009 (3)	0.004 (3)	−0.003 (3)
O1	0.044 (3)	0.070 (5)	0.040 (3)	−0.005 (3)	0.021 (3)	0.000 (3)
O2	0.060 (4)	0.079 (5)	0.031 (3)	0.002 (3)	0.023 (3)	0.000 (3)
O3	0.065 (4)	0.081 (5)	0.032 (3)	−0.035 (4)	0.004 (3)	−0.012 (3)
O4	0.046 (3)	0.090 (5)	0.028 (3)	−0.012 (3)	0.013 (3)	−0.001 (3)
O5	0.054 (4)	0.075 (5)	0.038 (3)	−0.008 (3)	0.020 (3)	0.005 (3)
BR1	0.0721 (8)	0.0855 (9)	0.0375 (5)	−0.0200 (6)	0.0007 (5)	−0.0053 (5)

### Geometric parameters (Å, º)

**Table:**

C1—C6	1.369 (10)	C9—H9	0.90 (5)
C1—C2	1.386 (10)	C10—C15	1.394 (9)
C1—H1	0.9300	C10—C11	1.404 (10)
C2—C3	1.383 (9)	C11—O5	1.362 (9)
C2—C7	1.479 (10)	C11—C12	1.379 (11)
C3—C4	1.385 (9)	C12—C13	1.388 (11)
C3—H3	0.9300	C12—H12	0.9300
C4—C5	1.399 (10)	C13—C14	1.385 (11)
C4—N1	1.454 (8)	C13—H13	0.9300
C5—N2	1.361 (9)	C14—C15	1.377 (10)
C5—C6	1.411 (9)	C14—BR1	1.899 (7)
C6—H6	0.9300	C15—H15	0.9300
C7—O2	1.201 (8)	C16—O5	1.428 (9)
C7—O1	1.346 (9)	C16—H16A	0.9600
C8—O1	1.437 (9)	C16—H16B	0.9600
C8—H8A	0.9600	C16—H16C	0.9600
C8—H8B	0.9600	N1—O3	1.221 (8)
C8—H8C	0.9600	N1—O4	1.240 (7)
C9—N3	1.277 (9)	N2—N3	1.371 (8)
C9—C10	1.464 (11)	N2—H2A	0.83 (6)
			
C6—C1—C2	121.8 (6)	C11—C10—C9	120.8 (7)
C6—C1—H1	119.1	O5—C11—C12	124.1 (7)
C2—C1—H1	119.1	O5—C11—C10	115.8 (7)
C3—C2—C1	118.1 (7)	C12—C11—C10	120.1 (7)
C3—C2—C7	121.8 (7)	C11—C12—C13	120.9 (8)
C1—C2—C7	120.0 (6)	C11—C12—H12	119.5
C2—C3—C4	120.2 (7)	C13—C12—H12	119.5
C2—C3—H3	119.9	C14—C13—C12	118.6 (8)
C4—C3—H3	119.9	C14—C13—H13	120.7
C3—C4—C5	122.8 (6)	C12—C13—H13	120.7
C3—C4—N1	115.5 (7)	C15—C14—C13	121.5 (7)
C5—C4—N1	121.7 (6)	C15—C14—BR1	119.1 (6)
N2—C5—C4	124.8 (6)	C13—C14—BR1	119.4 (6)
N2—C5—C6	119.6 (7)	C14—C15—C10	120.0 (7)
C4—C5—C6	115.5 (6)	C14—C15—H15	120.0
C1—C6—C5	121.6 (7)	C10—C15—H15	120.0
C1—C6—H6	119.2	O5—C16—H16A	109.5
C5—C6—H6	119.2	O5—C16—H16B	109.5
O2—C7—O1	123.1 (7)	H16A—C16—H16B	109.5
O2—C7—C2	125.0 (8)	O5—C16—H16C	109.5
O1—C7—C2	111.9 (6)	H16A—C16—H16C	109.5
O1—C8—H8A	109.5	H16B—C16—H16C	109.5
O1—C8—H8B	109.5	O3—N1—O4	122.3 (6)
H8A—C8—H8B	109.5	O3—N1—C4	119.6 (6)
O1—C8—H8C	109.5	O4—N1—C4	118.0 (6)
H8A—C8—H8C	109.5	C5—N2—N3	119.2 (6)
H8B—C8—H8C	109.5	C5—N2—H2A	118 (4)
N3—C9—C10	119.5 (7)	N3—N2—H2A	122 (4)
N3—C9—H9	119 (4)	C9—N3—N2	116.3 (6)
C10—C9—H9	121 (4)	C7—O1—C8	116.8 (6)
C15—C10—C11	118.9 (7)	C11—O5—C16	118.2 (7)
C15—C10—C9	120.3 (7)		

### Hydrogen-bond geometry (Å, º)

**Table:**

*D*—H···*A*	*D*—H	H···*A*	*D*···*A*	*D*—H···*A*
N2—H2*A*···O4	0.83	2.03	2.635 (3)	129
C3—H3···O1	0.93	2.39	2.712 (4)	100
C6—H6···N3	0.93	2.40	2.731 (4)	101
C6—H6···O4^i^	0.93	2.59	3.444 (5)	152
C15—H15···O4^i^	0.93	2.46	3.358 (4)	161

Symmetry code: (i) *x*−1/2, −*y*−1/2, *z*−1/2.

## References

[bb1] Banerjee, S., Mondal, S., Chakraborty, W., Sen, S., Gachhui, R., Butcher, R. J., Slawin, A. M. Z., Mandal, C. & Mitra, S. (2009). *Polyhedron*, **28**, 2785–2793.

[bb2] Belskaya, N. P., Dehaen, W. & Bakulev, V. A. (2010). *Arch. Org. Chem.* **1**, 275–332.

[bb3] Bernstein, J., Davis, R. E., Shimoni, L. & Chang, N.-L. (1995). *Angew. Chem. Int. Ed. Engl.* **34**, 1555–1573.

[bb4] Bruker (2008). *APEX2* and *SAINT*. Bruker AXS Inc., Madison, Wisconsin, USA.

[bb5] Corey, E. J. & Enders, D. (1976). *Tetrahedron Lett.* **17**, 11–14.

[bb6] Cortés, E., Abonía, R., Cobo, J. & Glidewell, C. (2013). *Acta Cryst.* C**69**, 754–760. 10.1107/S0108270113013590 23832037

[bb7] Dey, D. & Chopra, D. (2017). *Acta Cryst.* B**73**, 781–793. 10.1107/S2052520617006643 28980982

[bb8] Groom, C. R., Bruno, I. J., Lightfoot, M. P. & Ward, S. C. (2016). *Acta Cryst.* B**72**, 171–179. 10.1107/S2052520616003954 PMC4822653 27048719

[bb9] Günes, B., Özbey, S. & Tezcan, H. (2003). *Anal. Sci.* **19**, 1091–1092. 10.2116/analsci.19.1091 12880102

[bb10] Levrand, B., Fiebera, W., Lehn, J.-M. & Herrmann, A. (2007). *Helv. Chim. Acta*, **90**, 2281–2314.

[bb11] Li, Y., Zhang, C. G., Cai, L. Y. & Wang, Z. X. (2013). *J. Coord. Chem.* **66**, 3100–3112.

[bb12] Li, L., Zhu, L., Chen, D., Hu, X. & Wang, R. (2011). *Eur. J. Org. Chem.* pp. 2692–2696.

[bb13] Luo, Y. H. & Sun, B. W. (2014). *Spectrochim. Acta Part A*, **120**, 381–388. 10.1016/j.saa.2013.10.036 24211619

[bb14] Luo, Y. H., Wu, G. G., Mao, S. L. & Sun, B. W. (2013). *Inorg. Chim. Acta*, **397**, 1–9.

[bb15] McKinnon, J. J., Spackman, M. A. & Mitchell, A. S. (2004). *Acta Cryst.* B**60**, 627–668. 10.1107/S0108768104020300 15534375

[bb16] Mufakkar, M., Tahir, M. N., Tariq, M. I., Ahmad, S. & Sarfraz, M. (2010). *Acta Cryst.* E**66**, o1887. 10.1107/S160053681002533X PMC3007545 21588223

[bb17] Narang, R., Narasimhan, B. & Sharma, S. (2012). *Curr. Med. Chem.* **19**, 569–612. 10.2174/092986712798918789 22204327

[bb18] North, A. C. T., Phillips, D. C. & Mathews, F. S. (1968). *Acta Cryst.* A**24**, 351–359.

[bb19] Rollas, S. & Küçükgüzel, S. G. (2007). *Molecules*, **12**, 1910–1939. 10.3390/12081910 PMC6149174 17960096

[bb20] Seth, S. K., Mandal, P. C., Kar, T. & Mukhopadhyay, S. (2011). *J. Mol. Struct.* **994**, 109–116.

[bb21] Shad, H. A., Tahir, M. N., Tariq, M. I., Sarfraz, M. & Ahmad, S. (2010). *Acta Cryst.* E**66**, o1955. 10.1107/S1600536810025882 PMC3007397 21588279

[bb22] Sheldrick, G. M. (2008). *Acta Cryst.* A**64**, 112–122. 10.1107/S0108767307043930 18156677

[bb23] Spackman, M. A. & Jayatilaka, D. (2009). *CrystEngComm*, **11**, 19–32.

[bb24] Spek, A. L. (2009). *Acta Cryst.* D**65**, 148–155. 10.1107/S090744490804362X PMC2631630 19171970

[bb25] Tahir, M. N., Tariq, M. I., Tariq, R. H. & Sarfraz, M. (2011). *Acta Cryst.* E**67**, o2377. 10.1107/S1600536811032958 PMC3200606 22065394

[bb26] Toledano-Magaña, Y., García-Ramos, J. C., Navarro-Olivarria, M., Flores-Alamo, M., Manzanera-Estrada, M., Ortiz-Frade, L., Galindo-Murillo, R., Ruiz-Azuara, L., Meléndrez-Luevano, R. M. & Cabrera-Vivas, B. M. (2015). *Molecules*, **20**, 9929–9948. 10.3390/molecules20069929 PMC6272681 26035095

[bb27] Uppal, G., Bala, S., Kamboj, S. & Saini, M. (2011). *Der. Pharma Chem.* **3**, 250–68.

[bb28] Vickery, B., Willey, G. R. & Drew, M. G. B. (1985). *Acta Cryst.* C**41**, 1072–1075.

[bb29] Wu, A., Senter, M. & Peter, D. (2005). *Nat. Biotechnol.* **23**, 1137–1146. 10.1038/nbt1141 16151407

[bb30] Xavier, A. J., Thakur, M. & Marie, J. M. (2012). *J. Chem. Pharm. Res.* **4**, 986–990.

